# Study on Performance and Engineering Application of Novel Expansive Superfine Cement Slurry

**DOI:** 10.3390/ma17225597

**Published:** 2024-11-15

**Authors:** Xiao Feng, Xiaowei Cao, Lianghao Li, Zhiming Li, Qingsong Zhang, Wen Sun, Benao Hou, Chi Liu, Zhenzhong Shi

**Affiliations:** 1 School of Transportation Engineering, Shandong Jianzhu University, Jinan 250101, China; 2022155138@stu.sdjzu.edu.cn (L.L.); 2023155138@stu.sdjzu.edu.cn (W.S.); 2023155140@stu.sdjzu.edu.cn (B.H.); 2024155112@stu.sdjzu.edu.cn (Z.S.); 2 Xuzhou Metro Group Co., Ltd., Xuzhou 221000, China; caoxiaowei@xzdtjt.com (X.C.); lizhiming@xzdtjt.com (Z.L.); 3 School of Civil Engineering, Shandong University, Jinan 250061, China; zhangqingsong@sdu.edu.cn (Q.Z.); 202420860@mail.sdu.edu.cn (C.L.); 4 Jinan Rail Transit Group Co., Ltd., Jinan 250000, China

**Keywords:** superfine cement, setting time, reinforcement strength, volume expansion, engineering application

## Abstract

Superfine cement is widely used in building reinforcement and repair, special concrete manufacturing, and environmental protection engineering due to its high toughness, high durability, good bonding strength, and environmental friendliness. However, there are some problems in superfine cement slurry, such as high bleeding rate, prolonged setting time, and consolidated body volume retraction. In this article, on the premise of using the excellent injectability of superfine cement slurry, the fluidity, setting time, reinforcement strength, and volume expansion rate of novel expansive superfine cement slurries with varying proportions were analyzed by adding expansion agent UEA, naphthalene-based water reducer FDN-C, and triisopropanolamine accelerating agent TIPA. The results show that under most mix ratios, the bleeding rate and fluidity of the novel superfine cement slurry initially increase and decrease with rising water-reducing agent dosage. The initial setting time generally decreases with accelerating agent dosage, reaching a minimum value of 506 min, representing a 33.68% reduction compared to the benchmark group (traditional superfine cement). Under normal conditions, the compressive strength of the net slurry consolidation body is positively correlated with expansion agent dosage, achieving maximum strengths of 8.11 MPa at three days and 6.93 MPa at 28 days; these values are respectively higher by 6.7 MPa and 2.6 MPa compared to those in the benchmark group. On the seventh day, the volume expansion rate of the traditional superfine cement solidified sand body ranges from −0.19% to −0.1%, while that for the corresponding body formed from the novel superfine cement is between 0.41% and 1.33%, representing a difference of 0.6–1.43%. After the on-site treatment of water and sand-gushing strata, the core monitor rate of the inspection hole exceeds 70%. The permeability coefficient of the stratum decreases to a range between 1.47 × 10^−6^ and 8.14 × 10^−6^ cm/s, resulting in nearly a thousandfold increase in stratum impermeability compared to its original state. Hence, the findings of this research hold practical importance for the future application of such materials in the development of stratum reinforcement or building repair.

## 1. Introduction

The stable progress of infrastructure construction in China has led to the emergence of projects such as highways, railways, and urban subways traversing the Quaternary sand layer. The loose structure and intricate seepage characteristics often result in disasters such as water gushing, sand collapse, and structural water leakage. Grouting emerges as the predominant technical solution to prevent and manage such disasters. This method involves injecting a solidifiable slurry into the crevices or voids of rock and soil masses to enhance their physical and mechanical properties. However, when grouting in medium-fine sand layers, conventional Portland cement presents issues related to its larger particle size and higher bleeding rate. In contrast, traditional superfine cement is associated with prolonged gel time and susceptibility to volume shrinkage of the solidified mass. Therefore, exploring novel superfine cement slurries suitable for grouting in medium-fine sand layers is of significant scientific importance.

Currently, numerous scholars have conducted significant experimental studies on the modification of superfine cement materials. Han Fenglei et al. [1] investigated the effects of the total volume fraction of SiO_2_ gas glue and sand particles at 60% on the flowability, dry density, water absorption, thermal conductivity, mechanical properties, and microstructure of superfine cement-based materials. The results showed that with the increase of aerogel content from 0% to 60%, the material fluidity decreased by 7.3–22.3%, and the dry density gradually decreased while the water absorption increased. Zhang Jianwu et al. [2] explored the influence and mechanism of soluble P_2_O_5_ on the early-age hydration behavior of superfine sulfoaluminate cement, finding that soluble P_2_O_5_ has a minor effect on setting time but can significantly increase the formation of early calcarenite. When the soluble P_2_O_5_ content was between 0% and 1.5%, the early strength of superfine sulfoaluminate cement-based double liquid grouting materials increased with the soluble P_2_O_5_ content. Zhao Weiquan et al. [3] investigated the pH value of superfine cement slurry at different ages under silica fume content conditions and a water–binder ratio set at 1.4. They determined that when silica fume content is more than 40%, SiO_2_ content is more than 50%, and the Ca/Si molar ratio of C-S-H gel is less than 0. 8, the pH value of the consolidation body is less than 11. Tongqiang Xiao et al. [4] to solve the problem of grouting reinforcement of micro-fractures surrounding rock in deep roadways, the influence characteristics of auxiliary materials and additives on slurry flow were analyzed, and the composition and proportion of superfine cement-based composite grouting materials were determined: superfine cement accounted for 89.4%, superfine coal ash accounted for 5%, ultrafine mineral powder accounted for 5%, naphthalene water reducing agent accounted for 3~5‰, and lignin sulfonate calcium accounted for 1~3‰. When the water-cement ratio is 1.0 and the water reducer content is 3 ‰, the slurry has the advantages of strong permeability, strong fluidity, and low water bleeding rate. Dongyue Zhang et al. [5] examined the effect of superfine cement content on the strength and microstructure characteristics of solidified materials through uniaxial compression tests, fragment fractal dimension analysis, mercury intrusion porosimetry experiments, and scanning electron microscopy. The results showed that the superfine cement content increased from 0% to 70% when the water–cement ratio was 0.45:1, the compressive strength of the grouted specimens increased from 16.7 MPa to 26.3 MPa, and the fractal dimension decreased from 1.8645 to 1.2301. Shengnan Xu et al. [6] investigated various methods, including mechanical grinding, sulfate activation, and alkali activation, to enhance the reactivity of mineral admixtures. They also analyzed the evolution process of cementitious materials from microstructure to macroscopic mechanical behavior using a laser granularity analyzer and scanning electron microscopy. It is concluded that under the condition that the compressive strength is not lower than that of the control group (without mineral admixture), the content of mineral admixture can be increased to 50%, 70%, and 90% after mechanical grinding, sulfate activation, and alkali activation, respectively. Hao Li et al. [7] utilized oil well cement and superfine cement as raw materials to produce a composite material with excellent compressive and tensile strength by incorporating green carbonation calcium and steel fibers. The strength test results on day 28 showed that the compressive and tensile strength of the material increased from 44 MPa to 74 MPa and from 3.6 MPa to 18.3 MPa compared with the material without the addition of micron-size green silicon carbide. Fei Wang et al. [8] developed the modified coral aggregate concrete, and the effect of using modified superfine cement on its workability and strength is investigated. Experimental results show that the strength of the modified coral aggregates cured for a short duration is slightly lower than that of unmodified coral aggregates, whereas that cured for 28 days is approximately 20% higher than that of unmodified coral aggregates. Longji Wu et al. [9] developed a novel polyacrylate emulsion-modified superfine cement slurry composed of superfine cement, polyacrylic ester butadiene, defoaming agent, and accelerating agent; they compared the workability, mechanical properties, and microstructure of modified superfine cement slurries with different proportions.

Meanwhile, considerable research has been conducted on treating sandy soil layers with superfine cement. Murat Mollamahmutoglu et al. [10] investigated the compressive strength, permeability, expansion potential, and compressibility of high plasticity clay modified by slag-based superfine cement. They observed that wet curing and air drying conditions enhanced the strength of high plasticity clay when treated with slag-based superfine cement while slightly reducing permeability, expansion potential, and compressibility. Xiaoli Liu et al. [11] performed a theoretical analysis on sleeve valve grouting in sandy soil stratum using the discrete element method to simulate the diffusion behavior of Bingham fluids under varying pressures and to establish the relationship between grouting pressure, diffusion radius, and initial fracture pressure. Wenshuai Li et al. [12] enhanced the samples’ early shear strength, microstructure, and slurry viscosity by incorporating different particle sizes of cement and nano-SiO_2_. Results showed that the optimal superplasticizer dose levels of 1.5% and 0.75% were recommended for superfine cement grouts with and without nano-SiO_2_ addition, respectively. Eybhan et al. [13] measured the viscosity, bleeding capacity, setting time, strength, and permeability of superfine cement and fine fly ash mixtures with varying water–cement ratios. Their findings indicated that adding fly ash can enhance the compressive strength of the slurry. Jian Zhang et al. [14] discussed the feasibility of silty fine sand as a substitute sand source for backfill grouting liquid, and research shows that the grouting liquid with the best performance has advantages in terms of 7 d and 28 d strength, bleeding rate, and cementation strength. The stability of this grouting liquid is good, and 44.6% of costs can be saved. Fei Ye et al. [15] investigated the engineering characteristics of wind and sand tunnels using the Shenmu No. 1 tunnel as a case study. They determined the proportion of superfine cement slurry and modified water glass slurry. Yuke Wang et al. [16] compared three traditional grouting materials with a self-developed permeable polymer, analyzing the slurry viscosity, setting time, the strength of the silty sand consolidation body, water stability, and permeability coefficient. Regarding water stability, the specimens reinforced with the permeable polymer showed a minimal reduction of 10.87% after 7 days of immersion, exhibiting excellent water stability. Ying Cui et al. [17] prepared high-performance grouting materials (DS slurry) for water-rich silty fine sand stratum using water glass as the base material and diacid ester as the curing agent. The reaction mechanism between the water glass and the curing agent was studied using infrared spectroscopy and scanning electron microscopy, concluding that DS grouting exhibits adjustable setting time, high strength, and good durability. Grzegorz Ludwik Golewski [18] proposed a most commonly used mineral additive (i.e., fly ash (FA)) combined with nano-silica (NS) as a partial substitute for ordinary Portland cement (OPC) binder. The study found that after adding 15% FA and 5% NS additives to the binder, all mechanical parameters of concrete increased by about 20%.

In the past, another slurry was often introduced to improve the performance of ultrafine cement slurry to form a two-liquid grouting mode. At the same time, the research content is less related to the volume change of net slurry solidified body and solidified sand body of ultrafine cement slurry. This study aims to tackle the challenges of high water bleeding rate, prolonged setting time, and susceptibility to volume shrinkage in traditional superfine cement slurry while ensuring excellent injectability in medium-fine sand layers. By incorporating expansion agent UEA, naphthalene-based water reducer FDN-C, and triisopropanolamine accelerating agent TIPA, the fluidity, gel time, reinforcement strength, and volume expansion rate of novel superfine cement slurries with varying proportions are analyzed. Subsequently, focusing on medium-fine sand, the research investigates the diffusion distance, reinforcement strength, and volume expansion rate of the novel superfine cement slurry within this stratum. Ultimately, the practical application has confirmed the engineering viability of the novel expansive superfine cement.

## 2. Materials and Methods

### 2.1. Raw Materials

The cement utilized in the experiment is superfine cement produced by Sino-German Xinya Factory in Jinan, China. The fineness is 800 mesh, the specific surface area is 800 m^2^/kg, and the average particle size is 5 μm. The main characteristics of 800-mesh superfine cement include high strength, low permeability, good durability, and environmental protection. Because of its small particle size and large specific surface area, the strength of ultrafine cement is higher than that of ordinary cement.

The expansion agent used was the UEA concrete expansion agent, produced by Huayu Building Materials Factory in Nanchang, China. The main components are calcium sulphoaluminate clinker, alunite, and gypsum. The appearance is a grayish-white powder, the specific gravity is 2.8–3.0, and the specific surface area is not less than 250 m^2^/kg. The main characteristics of UEA concrete expansion agents include preventing shrinkage cracking, generating chemical pressure, low dosage, and low alkali content. For example, when the outside completely constrains the mortar consolidation body mixed with the UEA expansion agent, its expansion force will act internally. Ettringite crystals fill the pores to form a very dense, non-shrinkage mortar.

The water-reducing agent used was naphthalene water-reducing agent FDN-C, which is produced by Shandong Wanshan Chemical Co., Ltd., Weifang, China. It is a yellowish powder with a density of 80 g/cm^3^, a purity of 99%, a chloride ion content of <0.5%, and a sulfate content of <18%. The main characteristics of naphthalene water-reducing agent FDN-C include solubility in water and stable physical and chemical properties. It also has high dispersion and low foaming, which can significantly improve the fluidity of concrete. The strength and early strength of concrete after adding FDN-C are remarkable; the strength of one day and three days is increased by 60–95%, and the strength of twenty-eight days is increased by 25–40%.

The rapid setting admixture used was the triisopropanolamine accelerating agent TIPA, produced by the Jinyu Chemical Plant in Dehui, China. The molecular formula is C_9_H_21_NO_3_, a white crystal with a density of 0.994 g/mL and a solubility of 83 g/100 mL in water at 20 °C. The characteristics of triisopropanolamine accelerator mainly include: (1) It can improve the density of concrete by inhibiting the occurrence of hydration reaction, thereby enhancing the strength and durability of concrete. (2) It can increase the ductility and tensile strength of concrete, make the concrete material more flexible, and reduce the occurrence of concrete cracks. (3) It can form a three-dimensional network structure during the hardening process of concrete to prevent cracks in the concrete during drying shrinkage.

### 2.2. Test Methods and Plan

**Figure 1:**
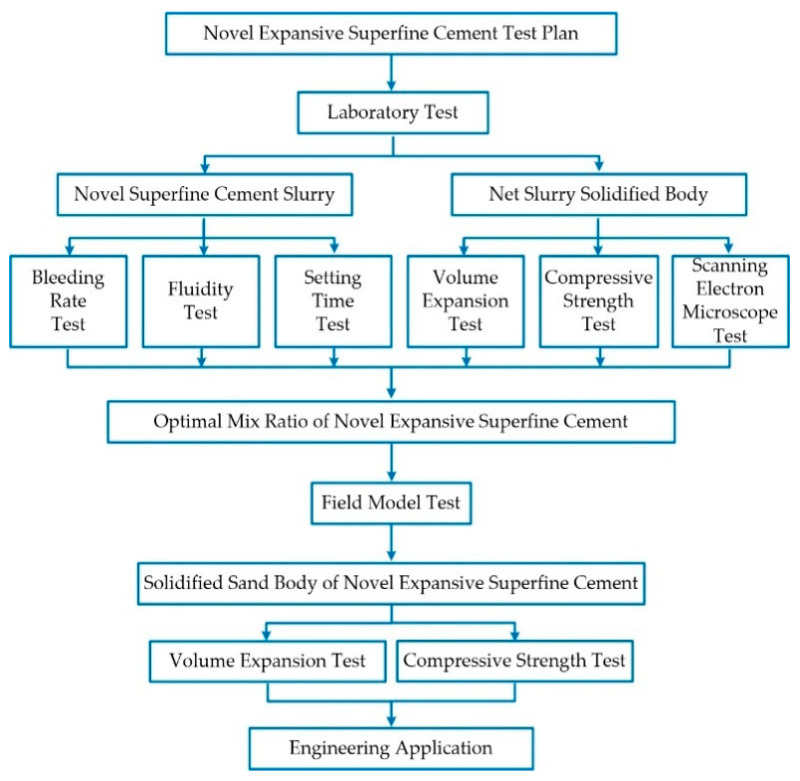
The test plan of novel expansive superfine cement.

The flowability of the slurry was evaluated using the XN-Φ36 mm × 60 mm × 60 mm Cement paste flow conical die, as described in the Testing Methods of Cement and Concrete for Highway Engineering [19]. The setting time of the slurry was determined with a Vicat apparatus, following the testing method outlined in Test Methods for Water Requirement of Normal Consistency, Setting Time, and Soundness of the Portland Cements [20]. The compressive strength of the consolidation body was assessed using a material strength comprehensive tester, the testing method specified in Test Method for Axial Compressive Strength of Cement Concrete Cylinder [21]. The expansion volume of the specimen was measured using a comparator, following the testing method outlined in the Test Method for Determining Expansive Ratio of Expansive Cement [22]. The test plan is shown in Figure 1.

## 3. Laboratory Test and Analysis

### 3.1. Orthogonal Experiment Design

Under the condition of a water–cement ratio of 1:1 (water 400 g, superfine cement 400 g), after conducting extensive research on the literature, the dosage ranges of the expansion agent UEA, naphthalene-based water reducer FDN-C, and triisopropanolamine accelerating agent TIPA were initially selected (are shown in Table 1). Following the concept and method of orthogonal experimental design, a three-factor, three-level experimental design was implemented (shown in Table 2).

### 3.2. Analysis of Bleeding Rate and Fluidity of Slurry

**Figure 2:**
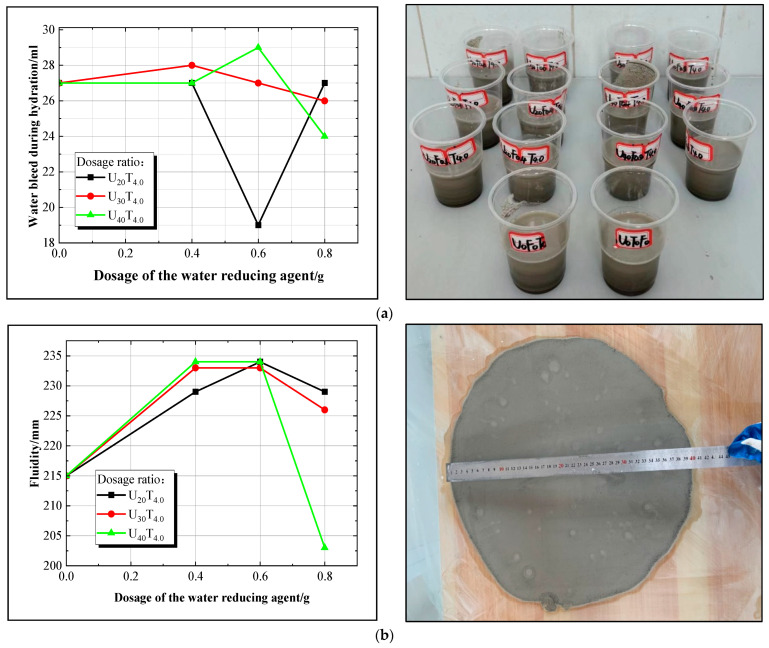
(**a**) Curve of variation in slurry water bleed; (**b**) curve of slurry fluidity variation.

The study aimed to investigate the influence of naphthalene sulfonate superplasticizer FDN-C on the bleeding rate and fluidity of novel superfine cement slurry. Data analysis was conducted using specific groups (1, 4, 7, 10, 13, 16, 19, 22, 25, and 28) as representatives. Detailed results are presented in Figure 2. In Figure 2, the expansion agent UEA, naphthalene-based water reducer FDN-C, and triisopropanolamine accelerating agent TIPA are represented as U, F, and T respectively. For example, U_30_F_0.6_T_2.6_ indicates that the UEA dosage is at a level of 30 g, while the dosages for FDN-C and TIPA are at levels of 0.6 g and 2.6 g, respectively; U_20_T_4.0_ indicates a UEA dosage of 20 g with varying dosages for FDN-C (ranging from 0 g to 0.8 g) and a TIPA dosage of 4 g.

Figure 2a demonstrates that the initial water bleed of the benchmark group (traditional superfine cement) is 27 mL. When considering the U_20_T_4.0_ ratio, the water bleed rate of the slurry initially decreases, then increases with the rise in water reducer dosage, reaching a minimum of 19 mL. For the U_30_T_4.0_ and U_40_T_4.0_ ratios, the water bleed rate of the slurry first increases, then decreases as the water reducer dosage increases, with minimum values of 26 mL and 24 mL, respectively. In Figure 2b, the initial flowability of the benchmark group is 215 mm. The flowability of the novel superfine cement slurry with U_20_T_4.0_, U_30_T_4.0_, and U_40_T_4.0_ ratios all exhibit an increasing trend followed by a decreasing trend with the increased water reducer dosage, peaking at 235 mm. Notably, the U_40_T_4.0_ ratio shows a significant decrease in flowability, reaching a minimum value of 203 mm, which is 5.6% lower than that of the benchmark group.

The analysis indicates that introducing a water reducer into the superfine cement slurry leads to the dispersion of superfine cement particles, which is attributed to the static repulsion induced by the water reducer. This dispersion improves the suspension stability of the cement particles, thereby reducing the bleeding rate of the slurry. Furthermore, the adsorption film formed by the water reducer on the surface of superfine cement particles demonstrates a favorable lubricating effect, effectively reducing inter-particle sliding resistance and enhancing slurry flowability [16,23]. However, studies have indicated that exceeding a certain threshold in water reducer dosage may decrease slurry flowability. In conclusion, the slurry ratio with a low bleeding rate and high fluidity was initially selected, meeting the requirements in groups 4, 7, 10, 13, 16, 19, 22, and 25.

### 3.3. Setting Time Analysis

**Figure 3:**
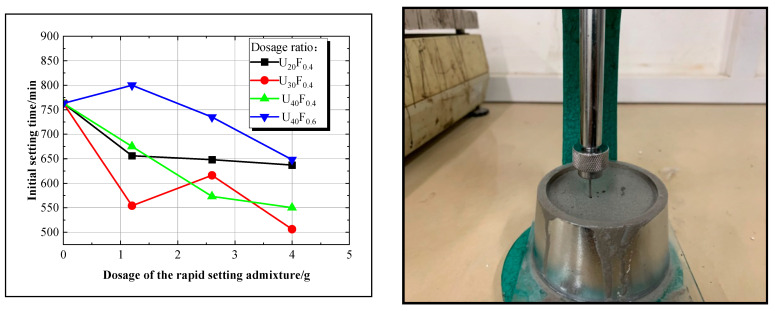
Curve of setting time and its experimental setup.

This study conducted a comparative analysis of the test results from groups 1, 2, 3, 4, 11–13, and 20–25 to investigate the impact of the accelerating agent TIPA on the setting time of the novel superfine cement slurry. Detailed findings are presented in Figure 3.

The analysis presented in Figure 3 illustrates a general reduction in the initial setting time as the dosage of the accelerating agent TIPA increases. Only the slurry with the U_40_F_0.6_T_1.2_ ratio exhibited an initial setting time that exceeded the benchmark group (763 min). The U_30_F_0.4_T_4.0_ ratio achieved the shortest initial setting time of 506 min, representing a 33.68% reduction compared to the benchmark group. Similarly, the U_40_F_0.4_T_4.0_ ratio reached a minimum initial setting time of 550 min, 27.92% shorter than the benchmark group value. Consequently, incorporating TIPA as a rapid-setting admixture significantly reduces the initial setting time of superfine cement slurry.

Analysis indicates that when the dosage ranges from 0.3% to 1.0%, triisopropanolamine (TIPA) diminishes the interfacial tension between superfine cement particles and water, accelerates the hydration process, and interacts with tricalcium aluminate (C_3_A) to facilitate the formation of calcium sulfoaluminate (AFt), ultimately reducing the setting time of the slurry [24,25,26,27]. Consequently, this study made a preliminary selection for the slurry mixture with an initial setting time shorter than the benchmark group, meeting requirements for groups 2–4, 11–13, 20–22, and 24–25.

### 3.4. Investigation of Volume Expansion and Compressive Strength of Net Slurry Consolidation Body

#### 3.4.1. Analysis of Volume Expansion of Net Slurry Solidified Body

**Figure 4:**
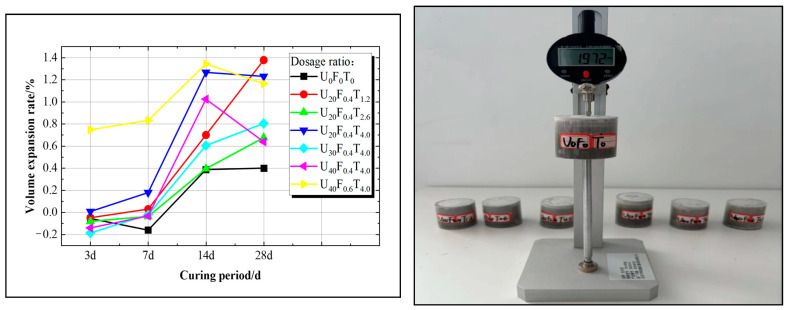
Expansion rate curve of net slurry solidified body and its experimental setup.

To address the issue of volume shrinkage in the later stages of the solidification body for net superfine cement slurry, varying dosages of expansion agent UEA were introduced. The expansion rates of specimens at the age of day 3, day 7, day 14, and day 28 for groups 1, 2, 3, 4, 13, 22, and 25 were determined using a comparator, as shown in Figure 4.

Figure 4 illustrates that the volume of the net slurry consolidation body in the benchmark group experienced a slight shrinkage (approximately −0.2%) from day 3 to day 7. In contrast, the volume of the net slurry consolidation body expanded significantly in the U_40_F_0.6_T_4.0_ and U_20_F_0.4_T_4.0_ groups. The volume of the other groups did not expand compared with the original volume, but compensation for previous volume shrinkage occurred. With the curing period extended, this study significantly enhanced the expansion performance of the novel superfine cement solidification body. For instance, on day 14, the volume expansion rate of U_20_F_0.4_T_4.0_ reached 1.2%, which was 0.8% higher than that of the benchmark group, and on day 28, the volume expansion rate of U_20_F_0.4_T_1.2_ was 1.4%, which was 1.0% higher than that of the benchmark group. Notably, when using an expansion agent dosage of 40 g (10%), the U_40_F_0.6_T_4.0_ ratio exhibited the highest volume expansion, with rates on day 3, day 7, day 14, and day 28 of 0.747%, 0.831%, 1.343%, and 1.164% respectively, which were 0.803%, 0.992%, 0.956%, and 0.528% higher than those of the benchmark group.

Analysis indicates that adding expansion agent UEA to superfine cement slurry enhances the compaction of the net slurry consolidation body by increasing the formation of calcium stones. The delayed formation of calcium scleral crystals mitigates the increase in capillary tension, leading to a significant increase in the volume expansion rate of the solidified body. Moreover, the generation of calcium stone is proportional to the content of the expansion agent, thereby correspondingly enhancing the effect of volume expansion.

#### 3.4.2. Analysis of Compressive Strength of Net Slurry Consolidation Body

**Figure 5:**
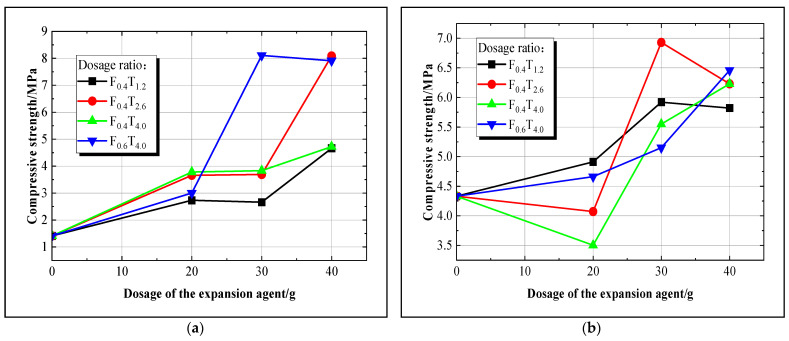
(**a**) Day 3 compressive strength curve of net slurry solidified body; (**b**) day 28 compressive strength curve of net slurry solidified body.

After completing the expansion volume test of the net slurry consolidation body for each group of new superfine cement, the compressive strength of the cylindrical specimens at day three and day 28 ages is determined using a comprehensive material strength tester, as shown in Figure 5.

According to Figure 5a, the compressive strength of the benchmark group was 1.41 MPa on the third day of curing age, and it shows a positive correlation between the compressive strength of the new superfine cement net slurry consolidation body and the dosage of the expansion agent UEA. When the ratio is U_30_F_0.6_T_4.0_, the third-day compressive strength of the net slurry solidified body peaks at 8.11 MPa. The trend of the F_0.4_T_2.6_ and F_0.4_T_4.0_ ratios are essentially similar. As shown in Figure 5b, the 28th-day compressive strength of the benchmark group was 4.33 MPa, while the compressive strength of the U_30_F_0.4_T_2.6_ ratio reached a peak of 6.93 MPa, demonstrating a 60% increase in strength compared to the benchmark group. In most cases, there is a positive correlation between expansion agent dosage and 28th-day compressive strength; however, under the specific condition with an expansion agent dosage of 20 g, the strengths of the F_0.4_T_2.6_ and F_0.4_T_4.0_ are 4.07 MPa and 3.50 MPa, respectively, which are 6% and 19.17% lower than the initial values, indicating that these ratios are suboptimal.

Analysis suggests that the UEA expansion agent contains active components, such as Al_2_O_3_ and CaSO_4_, which react with the Ca(OH)_2_ in the superfine cementitious material to generate calcium stone (AFt). This process accelerates the early hydration rate of the new superfine cement slurry, increasing the internal stress of the slurry and enhancing the compressive strength of the consolidation body [28,29,30].

Based on a comprehensive analysis of the indicators, groups 4, 13, 22, and 25 have been selected as the optimal mix ratios for subsequent field testing to assess the actual grouting effect of the new expansion superfine cement material.

### 3.5. Scanning Electron Microscope Analysis of Net Slurry Consolidation Body

**Figure 6:**
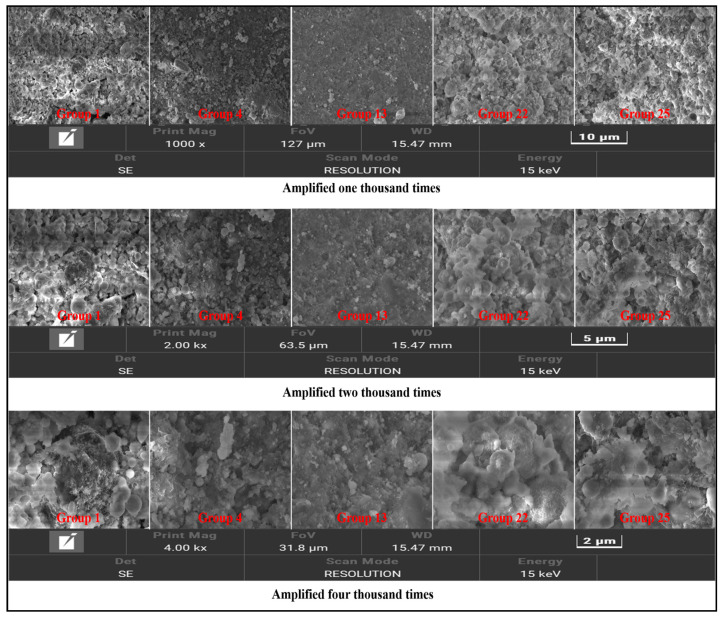
The microstructure of each group under different magnifications.

**Figure 7:**
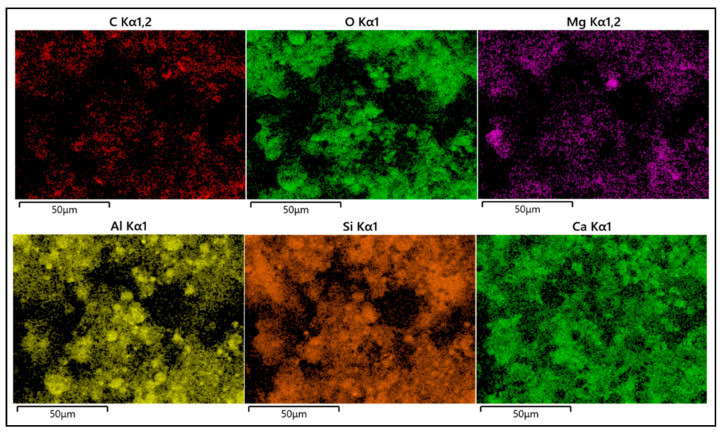
Elemental analysis of No. 22.

Group 1, Group 4, Group 13, Group 22, and Group 25 specimens were cured for seven days and dried at 20~25 °C for one day to make specimens with side lengths < 8 mm and thicknesses < 5 mm. The microstructure was analyzed by scanning electron microscopy (SEM), as shown in Figure 6. Due to space limitations, only the element analysis diagram of Group 22 is shown, as shown in Figure 7.

Figure 6 and Figure 7 show that Group 1 is the microstructure of a pure cement consolidation body. The matrix contains spherical condensation hydration products, which are considered to be C-S-H gel. The loose gel structure is due to the consolidation body’s high porosity under a high water–cement ratio, which decreases strength.

With the increase of the content of the expansion agent and accelerator, the volume of hydration gel decreases, but the number increases and the matrix is denser. On the one hand, the calcium sulphoaluminate clinker in the expansive agent can be rapidly hydrated, generating many hydration products while consuming calcium hydroxide, accelerating the hydration reaction rate of Portland cement. On the other hand, the accelerator triisopropanolamine can accelerate the dissolution of aluminum-containing minerals, significantly promote the hydration process, increase the formation of hydration products, and help improve the microstructure’s compactness. When the expansion agent content increases to 10%, the volume of the hydration product increases, the gap between the products cannot be effectively filled, and the compactness of the matrix decreases; this is attributed to the excessive expansion agent (effective components such as calcium sulphoaluminate clinker and gypsum) participating in the reaction. The matrix is subjected to the expansion stress of the excessive expansion product, and defects such as voids appear inside, resulting in a loose structure.

After the content of the naphthalene water-reducing agent increases, the bleeding rate of the material increases, the actual water–cement ratio decreases, and the free water amount involved in the hydration reaction decreases. Compared with Group 22, the internal product structure of Group 25 changed from gel to lamellar, which improved the pores. However, the hydration products could not fill the whole matrix, resulting in more structural voids and lower compactness.

## 4. Field Model Test

### 4.1. Test Materials

Sand samples near Xindong Station in Jinan were utilized as test objects for field model grouting tests. Various graded particle sizes were obtained through sieving tests, and the fineness modulus was calculated according to Formula (1). The average value was determined through three calculations, leading to the classification of the field sample as medium sand, as shown in Table 3.

(1) $$M_{X}=\frac{(A2+A3+A4+A5+A6)-5A1}{100-A1}$$

A_1_, A_2_, A_3_, A_4_, A_5_, and A_6_ represent the cumulative percentages of sieve residue for 4.75 mm, 2.36 mm, 1.18 mm, 0.60 mm, 0.30 mm, and 0.15 mm sieves, respectively.

### 4.2. Model Test Scheme

The field model grouting tests were conducted using the optimal mix ratio determined from laboratory experiments to investigate the diffusion effect and reinforcement performance of a novel expansion superfine cement slurry in sand layers. The experimental materials included ordinary Portland cement, 800-mesh superfine cement, and novel expansion superfine cement (Groups 4, 13, 22, and 25), with water–cement ratios designed at 1:1, 1.1:1, and 1.2:1 respectively, as shown in Table 4. The grouting final pressure was set at 2.0 MPa to analyze the slurry’s diffusion distance, sand body reinforcement strength, and volume expansion rate under varying conditions. The test scheme of the field model is shown in Table 5.

Seamless steel pipes were modified with a diameter of Φ 108 mm and a length of 1~2 m. One end was designed as the slurry inlet with a pressure gauge installed and the other as the slurry outlet with a pressure relief valve installed. Additionally, slurry outlets were set at intervals on the side wall of the seamless steel pipe, each equipped with a one-way valve.

### 4.3. Experimental Process

First, a layer of mold release agent was uniformly applied on the inner wall of the seamless steel pipe. Subsequently, the initial mass of the steel pipe was measured, and the steel pipe was then filled with on-site sand samples five times, with compaction performed after each filling. The filling mass of the sand sample was controlled to be 16.42 kg. Next, a water pressure test was conducted to evaluate the test device’s sealing performance and determine the sand sample’s initial permeability coefficient. Finally, a slurry was prepared based on the designed ratios and used to conduct field model grouting tests on the sand samples.

### 4.4. Test and Analysis of Compressive Strength of Solidified Sand Body

The solidified sand bodies resulting from penetration grouting in each group were numbered sequentially from the inlet to the outlet, the slurry diffusion distance was measured, and the solidified sand bodies were allowed to cure for a specified age of 7 days before conducting the unconfined compressive strength test. For solidified sand bodies with length–diameter ratios not equal to 2, conversion was carried out according to Formula (2).

(2) $$\sigma_{C}=\frac{8\sigma_{L}}{7+2\frac{D}{H}}$$

where *σ_c_* represents the specimen’s uniaxial compressive strength (MPa) with the length-diameter ratio of 2, while *σ_L_* denotes the uniaxial compressive strength (MPa) of non-standard specimens; additionally, D refers to the diameter (cm), and H refers to the height (cm) of the samples.

#### 4.4.1. Strength Analysis of Cement-Solidified Sand Bodies with Varying Particle Sizes

**Figure 8:**
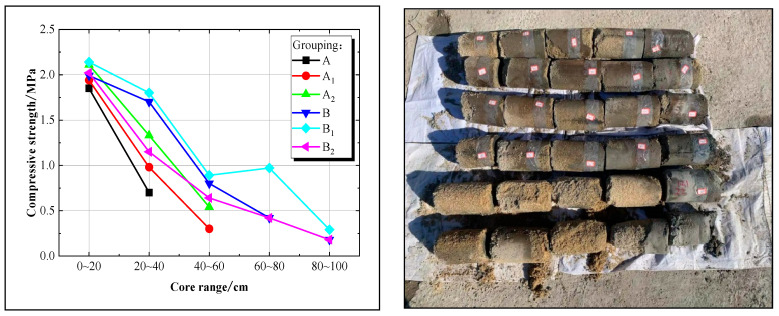
The compressive strength curve of the solidified sand body with ordinary Portland cement and 800-mesh superfine cement.

A comparative analysis was conducted utilizing ordinary Portland cement and 800-mesh superfine cement to evaluate the strength of the solidified sand body, with the test results illustrated in Figure 8.

As illustrated in Figure 8, it is evident that the diffusion range of ordinary Portland cement in Group A is confined to 20–40 cm, whereas Groups A_1_ and A_2_ exhibit a more comprehensive diffusion range of 40–60 cm. Furthermore, the 800-mesh superfine cement in Group B demonstrates a spreading distance of 60–80 cm, while Groups B_1_ and B_2_ can spread up to 80–100 cm. Analysis indicates that the spreading distance of the slurry is correlated with its injectability; specifically, higher water–cement ratios or smaller cement particle sizes result in increased spreading distances.

In the 20–40 cm section, Group B_1_ exhibits the highest compressive strength at 1.8 MPa, followed by Group B (1.7 MPa), Group A_2_ (1.33 MPa), Group B_2_ (1.15 MPa), Group A_1_ (0.98 MPa), and Group A (0.7 MPa). In the 40–60 cm section, Group B_1_ demonstrates a maximum compressive strength of 0.89 MPa, followed by Group B (0.8 MPa), Group B_2_ (0.64 MPa), Group A_2_ (0.54 MPa), and Group A_1_ (0.3 MPa). Comparison of Group B_1_ with Group B, or comparison of Groups A_2_, A_1_, and A under the same type of cement but different water–cement ratios, reveals that a higher water–cement ratio enhances injectability, retains more cement particles, and increases the strength of the solidified sand body. In comparison of Group B_1_ with Group A_1_, or comparison of Group B with Group A, it is concluded that at the same water–cement ratio but different cement types, smaller cement particle size leads to improved slurry injectability and higher retention of cement particles in the sand medium enhances the strength of the solidified sand body.

Consequently, injectability is directly related to the slurry’s diffusion distance and the strength of the solidified sand body. A higher water–cement ratio or smaller cement particle size results in a longer diffusion distance for the slurry. Simultaneously, maintaining a greater mass of cement within a specific diffusion range in the sand stratum leads to increased strength of the solidified sand body.

#### 4.4.2. Strength Analysis of Novel Superfine Cement-Solidified Sand Bodies with Varying Ratios

**Figure 9:**
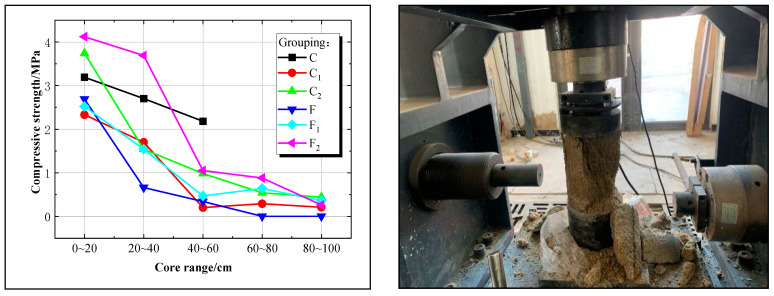
Reinforcement strength curve of novel superfine cement with different proportions.

The solidified sand body strengths of U_20_F_0.4_T_4.0_ and U_40_F_0.6_T_4.0_ were selected for comparative analysis to study the impact of the novel expansion superfine cement reinforcement sand medium. The experimental results are depicted in Figure 9.

Figure 9 illustrates that the diffusion distance of Group C is only 40–60 cm, whereas other groups exhibit a diffusion distance of 80–100 cm. Along the slurry’s diffusion distance, the overall trend in reinforcement strength of the solidified sand body decreases from Group F_2_ (4.12–0.26 MPa) to Group C (3.19–2.18 MPa), followed by Group C_2_ (3.74–0.44 MPa), Group F_1_ (2.52–0.36 MPa), Group C_1_ (2.33–0.21 MPa), and finally, Group F (2.69–0.34 MPa).

When utilizing the U_40_F_0.6_T_4.0_ mixing ratio, it was observed that Group F_2_ exhibited the highest strength, followed by Group F_1_, and then Group F showed the lowest strength. This indicates that under equivalent admixture dosage, a higher water–cement ratio in the slurry leads to increased grouting in the sand and retention of more cement particles within the sand, resulting in enhanced strength of the solidified sand body. In contrast, for the U_20_F_0.4_T_4.0_ mixing ratio, Group C demonstrated greater strength than both Group C_2_ and C_1_ due to its limited diffusion distance of only 40–60 cm; this results in most cement particles accumulating and being compressed within this range, thereby contributing to higher solidified sand body strength.

Furthermore, the comparison between the strengthening effect of Group F_2_ (U_40_F_0.6_T_4.0_) and Group C_2_ (U_20_F_0.4_T_4.0_), as well as Group F_1_ and Group C_1_, under identical water–cement ratio conditions reveals that the dosage of expansion agent exerts a primary influence on the solidified sand body’s strengthening effect, followed by the water reducer. The analysis indicates that the expansion agent and water reducer alter the hydration process of superfine cement particles, with a higher quantity of expansion agent resulting in an enhanced strengthening effect in the new slurry.

### 4.5. Investigation of the Solidified Sand Body Expansion Rate

**Figure 10:**
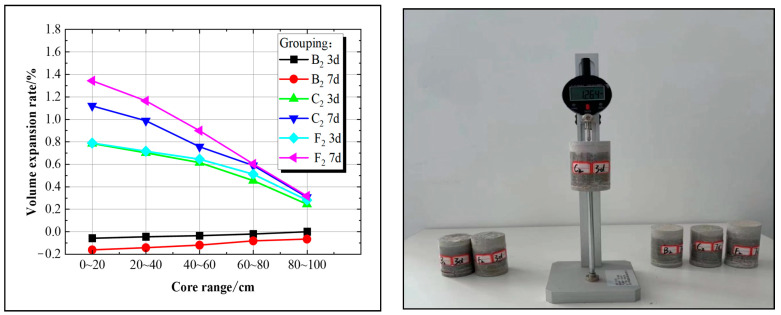
Analysis of solidified sand body expansion rate and its experimental setup.

After curing the solidified sand bodies in each group for 3 and 7 days, the volume of the solidified sand bodies was measured using a comparator. For instance, with a water–cement ratio of 1.2:1, Group B_2_ was utilized as the control group, while Groups C_2_ and F_2_ were designated experimental groups for comparative analysis. Detailed experimental results can be found in Figure 10.

Figure 10 demonstrates that, within the slurry diffusion range, the volume of net superfine cement solidification sand bodies exhibits shrinkage over time. For instance, on day 3, the volume expansion rate of the solidified sand bodies ranged from −0.04% to −0.02%, while on day 7, it ranged from −0.19% to −0.1%, indicating significant volumetric contraction. The shrinkage rate of the solidified sand body at the leading edge of the diffusion distance exceeds that at the trailing edge. For the novel expansion superfine cement material, within the slurry diffusion range, the volume of the solidified sand body expanded with age. In Group F_2_, the volume expansion rate ranged from 0.32% to 0.8% on day 3 and from 0.41% to 1.33% on day 7, indicating a significant increase in volume over time. Therefore, it is evident that the expansion performance of the novel superfine cement surpasses that of traditional superfine cement.

By the third day of curing, Group F_2_ exhibited a higher volume expansion rate than Group C_2_, ranging from 0% to 0.03%. However, by the 7th day of curing age, the disparity in volume expansion rates between the two groups had widened to 0.03%–0.23%. Analysis suggests that the content of the expansion agent in Group F_2_ (U_40_F_0.6_T_4.0_) was double that of Group C_2_ (U_20_F_0.4_T_4.0_), resulting in a significantly higher volume expansion degree for Group F_2_ compared to Group C_2_; however, this volume change primarily occurs during later stages due to variations in UEA expansion agent.

## 5. Engineering Application

### 5.1. Engineering Overview

In the Tianyuan Avenue comprehensive pipe gallery project at NK2+372–NK2+441.8 in Jinan East Railway Station, a water and sand gushing disaster occurred at the bottom of the pit. The initial remediation plan involved:

1. Utilizing drainage wells with a 700 mm diameter and a minimum depth of 15 m within the confined aquifer, spaced 5 m apart;
2. Reinforcing the pit bottom with double-pipe high-pressure jet grouting utilizing an 800 mm diameter and 550 mm spacing to reach 8.6 m below the pit bottom.

**Figure 11:**
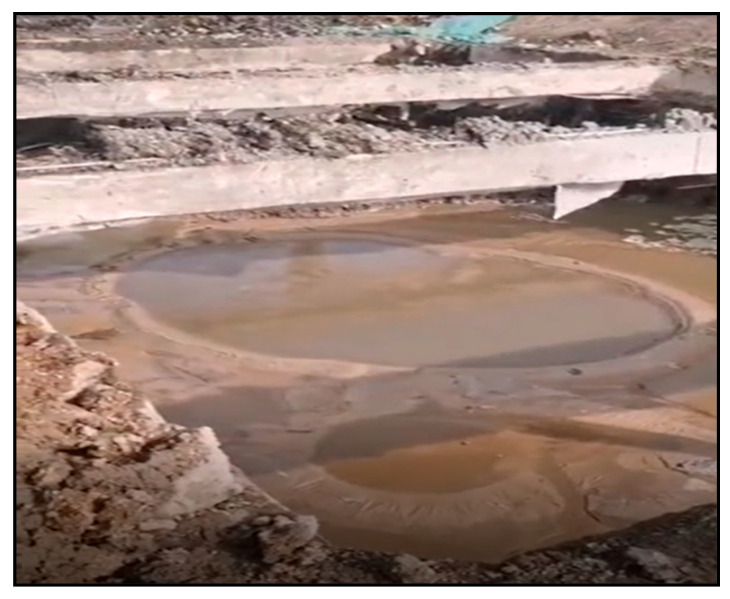
Water and sand gushing in the foundation pit.

Despite these measures, significant water seepage emerged from multiple points at the bottom of the pit when excavation reached approximately 4 m for the pipe gallery foundation, as shown in Figure 11.

A comprehensive approach involving systematic grouting reinforcement and waterproofing treatment was implemented to address the problem of water and sand gushing into the foundation pit. Initially, grouting holes were strategically positioned around the perimeter of the foundation pit to establish a waterproof curtain, effectively obstructing water pathways surrounding the foundation pit. Subsequently, evenly spaced grouting holes were installed within the foundation pit to seal off water channels and fractures in shallow strata, thereby enhancing the soil layers’ waterproofing capacity at the base of the excavation. Finally, structural reinforcement was designed at 33 m to revamp a deep gravel aquifer and mitigate pressure-induced water seepage.

### 5.2. Grouting Pressure and Diffusion Distance

The slurry diffusion distance represents the maximum distance that the slurry can migrate within a given time and pressure and is commonly used for evaluating grouting effectiveness. The grouting pressure is a critical factor influencing slurry diffusion distance. Excessive or inadequate grouting pressure may compromise reinforcement efficacy. Therefore, the permissible grouting pressure P_e_ can be calculated according to Formula (3):

(3) $$P_{e}=C(0.75T+Kh\lambda )\times {100\mathrm{kN}/m}^{3}$$

where *P*_e_ represents the allowable grouting pressure (KPa); *C* is a coefficient associated with the grouting sequence, taking on values of 1 for the first hole, 1.25 for the second hole, and 1.5 for the third hole; T denotes the thickness of the foundation cover layer (m); K is a coefficient related to the grouting method, taking on values of 0.5 for top-down grouting and 0.6 for bottom-up grouting; h represents the depth from ground level to the grouting section (m); and *λ* is a coefficient associated with strata properties, which can be selected within a range of 0.5 to 1.5 based on site conditions—lower values are chosen for loose and permeable structures while higher values are chosen otherwise.

The design and treatment depth of the strata in the pipe gallery foundation pit was 33 m, with drilling divided into three sections for progressive grouting from top to bottom. Specifically, the treatment depths were 0–13 m for the first section, 13–23 m for the second section, and 23–33 m for the third section. Substituting C = 1, T = 33 m, K = 0.5, h = 13 m, λ = 1 into Equation (3) yields an allowable grouting pressure of P_e1_=3.1 MPa for the first section; similarly, calculated values result in P_e2_ = 3.6 MPa for the second section and P_e3_ = 4.1 MPa for the third section.

According to Darcy’s law, q=KiA. When r = r_0_, h = H; r = r_1_, h = h_0_, this relationship can be derived:

(4) $$H-h_{0}=h_{1}=\frac{\beta q}{2\pi aK}\ln\frac{r_{1}}{r_{0}}$$

(5) $$q=\frac{2\pi aKh_{1}}{\beta \ln\frac{r_{1}}{r_{0}}}$$

Given these, $Q=\pi r_{1}^{2}an$ and Q=qt are then derived from Formula (5)

(6) $$r_{1}=\sqrt{\frac{2Kh_{1}t}{n\beta \ln(\frac{r_{1}}{r_{0}})}}$$

where *q* represents the grouting volume per unit time (cm³/s); *K* denotes the formation permeability coefficient, ranging from 1.20 × 10^−3^ to 6.0 × 10^−3^ cm/s; *a* is the permeation area (cm^2^); *r*_1_ stands for the diffusion radius of the slurry (cm); *r*_0_ is defined as the radius of the grouting pipe, set at 5 cm; *H* signifies the sum of groundwater head and grouting pressure head; h_0_ indicates the groundwater pressure head (cm); h_1_ refers to the allowable grouting pressure head, set between 31,000 cm and 41,000 cm; *β* represents the ratio of slurry viscosity to water viscosity, with a dynamic viscosity range of 1.77–2.26 mPa·s for novel superfine cement slurry and 1.0 mPa·s for water; a denotes the treatment section length (cm), set at 1000 cm; n stands for the formation porosity, taken as 0.3; *Q* represents slurry flow rate (cm^3^/s); and t denotes grouting time, set between 5 and 10 min.

According to Formula (6), the diffusion distance of the novel superfine cement slurry in engineering applications ranges from 104 cm to 361 cm. Moreover, with an allowable grouting pressure set at 2.0 MPa, that is, the allowable grouting pressure water head is set to 20,000 cm, the diffusion distance of the novel superfine cement slurry was estimated to be between 86 cm and 96 cm, which aligns closely with the actual diffusion distance observed in field model grouting tests.

### 5.3. Inspection and Evaluation of Grouting Effect

#### 5.3.1. Inspection Hole Coring and Evaluation

**Figure 12:**
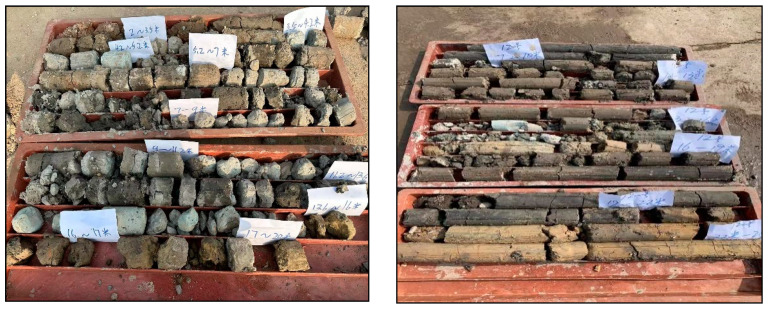
Cores from inspection holes 2 and 12.

Numerous inspection holes were constructed in the control area to visually and effectively demonstrate the impact of grouting reinforcement, and cores were extracted. Distinct grouting veins are discernible in cores, as shown in Figure 12.

Based on the integrity of the rock cores, a favorable overall reinforcement effect is indicated following the injection of the novel superfine cement slurry into the test area, with a core monitor rate exceeding 70% (the core monitor rate is the ratio of the core length to the footage length in a drilling process).

#### 5.3.2. Drilling Variable Head Seepage Test

Through the variable head penetration test, the permeability coefficient of the stratum is calculated according to the relationship between the head drop rate and time in the borehole to evaluate the reinforcement of the bottom layer. Detailed information on the borehole variable head seepage test can be found in Table 6.

The permeability coefficients of each inspection hole range from 1.47 × 10^−6^ cm/s to 8.14 × 10^−6^ cm/s, indicating relatively low values. The permeability of the stratum after grouting decreased by nearly three orders of magnitude compared to the pre-grouting value, which ranged from 1.20 × 10^−3^ cm/s to 6.0 × 10^−3^ cm/s.

#### 5.3.3. Transient Electromagnetic Detection

**Figure 13:**
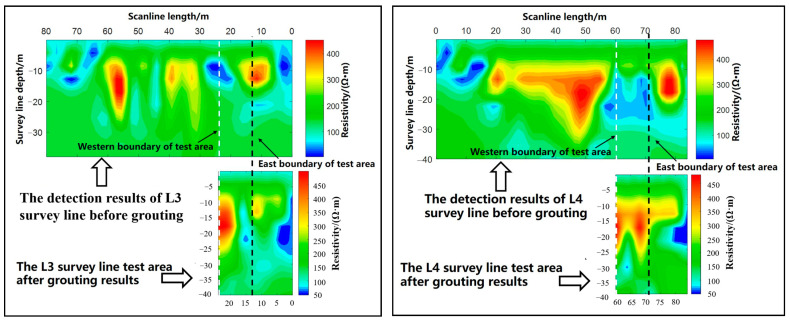
Comparison of L3 and L4 test lines pre- and post-grouting.

Transient electromagnetic methods utilize a non-grounded return line for transmitting pulse current underground. This induces eddy currents in low-resistivity subsurface media under the influence of the pulsed magnetic field, leading to the generation of a secondary magnetic field. Through post-power-off observation using receiving instruments and coils, an analysis of the characteristics and distribution of this secondary field enables the determination of geological body distribution patterns, as shown in Figure 13.

By comparing the L3 survey line before and after grouting, it is evident that there was a significant alteration in ground resistivity following the grouting process. The previously low-resistance area transformed into a regional high-resistance zone, indicating successful grouting. Similarly, comparing the L4 survey line before and after grouting, it is apparent that a substantial low-resistance area existed before grouting, suggesting pronounced water enrichment. Post-grouting, this low-resistance region has essentially disappeared, due to the evident filling and consolidation effects of the slurry.

#### 5.3.4. Excavation of Foundation Pit

**Figure 14:**
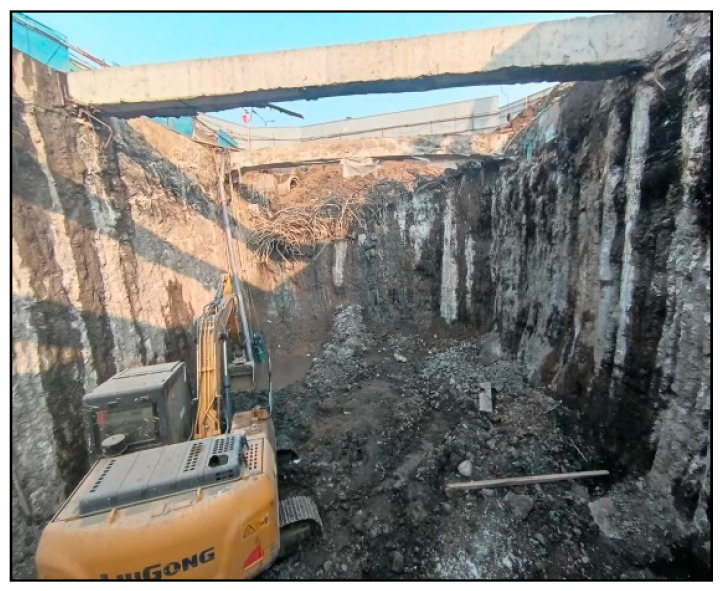
Excavation of foundation pit.

The comprehensive pipe gallery foundation pit was excavated following treatment with the novel superfine cement slurry. The on-site excavation situation demonstrated excellent integrity of the foundation pit sidewall, a significant increase in bottom layer strength post-grouting, and an absence of water channels or seepage cracks at the base of the pit, ensuring safe excavation of the foundation pit, as shown in Figure 14.

## 6. Conclusions

1. In most mix ratios, the bleeding rate and fluidity of the novel superfine cement slurry initially increase and then decrease with rising water-reducing agent dosage, reaching minimum bleeding water of 19 mL and maximum fluidity of 235 mm. The initial setting time generally decreases with increasing accelerating agent dosage, reaching a minimum value of 506 min, representing a 33.68% reduction compared to the benchmark group.
2. Between day 3 and day 7, solidified bodies formed from the novel superfine cement slurry are initially unchanged but demonstrate significant expansion as curing progresses. The volume expansion rate reached 1.4% on day 28, 1.0% higher than that of the benchmark group. Under normal conditions, the compressive strength of the net slurry consolidation body is positively correlated with expansion agent dosage, achieving maximum strengths of 8.11 MPa at 3 days and 6.93 MPa at 28 days; these values are respectively higher by 6.7 MPa and 2.6 MPa compared to those in the benchmark group.
3. On the 7th day, the volume expansion rate of the traditional superfine cement solidified sand body is −0.19%–−0.1%, while for the corresponding body formed from the novel superfine cement is 0.41%–1.33%; the expansion effect is significant. Under consistent water–cement ratio conditions, expansion agent dosage primarily influences the strength of solidified sand bodies, followed by the water-reducing admixture. However, the volume changes of solidified sand bodies resulting from variations in expansion agent dosage primarily manifest during later curing stages.
4. After the on-site treatment of water and sand-gushing strata, the core recovery rate of the inspection hole exceeded 70%. The permeability of the stratum after grouting decreased by nearly three orders of magnitude compared to the pre-grouting value, ranging from 1.47 × 10^−6^ cm/s to 8.14 × 10^−6^ cm/s. No water inrush occurred during the foundation pit excavation, demonstrating that the grouting performance is commendable.

## Figures and Tables

**Table 1:** Orthogonal test design table.

Level	Factor		
	UEA (Expansion Agent)/g	FDN-C (Water Reducer)/g	TIPA (Accelerating Agent)/g
1	20 (5%)	0.4 (0.1%)	1.2 (0.3%)
2	30 (7.5%)	0.6 (0.15%)	2.6 (0.65%)
3	40 (10%)	0.8 (0.2%)	4 (1%)

Note: Percentages in parentheses represent each admixture’s dosage relative to cement mass (400 g).

**Table 2:** Experiment scheme.

NO.	U/g	F/g	T/g	NO.	U/g	F/g	T/g	NO.	U/g	F/g	T/g
1	0	0	0	11	30	0.4	1.2	20	40	0.4	1.2
2	20	0.4	1.2	12			2.6	21			2.6
3			2.6	13			4.0	22			4.0
4			4.0	14		0.6	1.2	23		0.6	1.2
5		0.6	1.2	15			2.6	24			2.6
6			2.6	16			4.0	25			4.0
7			4.0	17		0.8	1.2	26		0.8	1.2
8		0.8	1.2	18			2.6	27			2.6
9			2.6	19			4.0	28			4.0
10			4.0								

Note: Expansion agent UEA, naphthalene-based water reducer FDN-C, and triisopropanolamine accelerating agent TIPA are designated U, F, and T, respectively.

**Table 3:** Grain grading of sand sample.

Sand Sample Number	Mass of Particles with Different Particle Sizes (g)							
	≥4.75 mm	4.75~2.3 mm	2.36~1.18 mm	1.18~0.6 mm	0.6~0.3 mm	0.3~0.15 mm	<0.15 mm	Fineness Modulus
1	0	7.6	88.4	98	111.5	137.2	48.8	3.04
2	0	1.7	89.8	133	123.2	107.2	40.7	3.14
3	0	1.9	66.1	137.9	128.2	106.3	45.6	3.0

**Table 4:** Experimental factors and parameters.

Level	Factor	
	Water–Cement Ratios	Different Materials
1	1:1	ordinary Portland cement
2	1.1:1	800-mesh superfine cement
3	1.2:1	U_20_F_0.4_T_4.0_ (Group 4)
4		U_30_F_0.4_T_4.0_ (Group 13)
5		U_40_F_0.4_T_0.4_ (Group 22)
6		U_40_F_0.6_T_4.0_ (Group 25)

**Table 5:** Field model test scheme.

NO.	W/C	Materials	NO.	W/C	Materials	NO.	W/C	Materials
A	1:1	ordinary Portland cement	A_1_	1.1:1	ordinary Portland cement	A _2_	1.2:1	ordinary Portland cement
B		800-mesh superfine cement	B_1_		800-mesh superfine cement	B _2_		800-mesh superfine cement
C		U_20_F_0.4_T_4.0_	C_1_		U_20_F_0.4_T_4.0_	C _2_		U_20_F_0.4_T_4.0_
D		U_30_F_0.4_T_4.0_	D_1_		U_30_F_0.4_T_4.0_	D _2_		U_30_F_0.4_T_4.0_
E		U_40_F_0.4_T_4.0_	E_1_		U_40_F_0.4_T_4.0_	E _2_		U_40_F_0.4_T_4.0_
F		U_40_F_0.6_T_4.0_	F_1_		U_40_F_0.6_T_4.0_	F _2_		U_40_F_0.6_T_4.0_

**Table 6:** Permeability coefficient of inspection hole.

Hole Number	1	3	7	8	9	10	11
Permeability Coefficient k 10^−6^ cm/s	1.90	2.14	5.49	8.14	6.89	1.47	7.30

Note: Surface water level is 2.0 m.

## Data Availability

The raw data supporting the conclusions of this article will be made available by the authors on request.
